# Mechanistic Investigation on the Regulation of FABP1 by the IL-6/miR-603 Signaling in the Pathogenesis of Hepatocellular Carcinoma

**DOI:** 10.1155/2021/8579658

**Published:** 2021-05-15

**Authors:** Ye-xin Lin, Xiong-bo Wu, Chu-wei Zheng, Qing-lin Zhang, Guo-qiang Zhang, Ke Chen, Qiang Zhan, Fang-mei An

**Affiliations:** Department of Gastroenterology, Wuxi People's Hospital Affiliated to Nanjing Medical University, Wuxi, Jiangsu 214023, China

## Abstract

**Background:**

Abnormal lipid metabolism is closely associated with the invasiveness and metastasis of cancer. Fatty acid-binding proteins (FABPs) play essential roles in lipid metabolism, and miRNAs can affect lipid metabolism by targeting FABPs. However, the exact mechanism is unknown.

**Methods:**

FABP1 expression in HCC tissues was analyzed by immunochemistry with tissue microarrays. The lipid content was detected by Oil Red O staining, and the interaction between FABP1 and free fatty acid (FFA) was studied by a labeling and tracking method. miRNA arrays were used to detect the expression of miRNAs in IL-6-stimulated HCC cells. miR-603 expression was verified by qPCR. The proteins were checked by Western blot analysis. Gain and loss function evaluation was assessed by lentivirus and miRNA mimic transfection in Huh-7 cells, while reactive oxygen species (ROS) were detected by fluorescence.

**Results:**

FABP1 expression was significantly decreased in approximately 90% (81/90) of HCC patients. FABP1 expression in adjacent tissues was closely associated with overall survival. Meanwhile, lipid was abundant in the adjacent tissues, yet significantly reduced in HCC tissues. FABP1 and FFA can promote each other for being uptaken by Huh-7 cells. FABP1 overexpression induced apoptosis and inhibited the proliferation, migration, invasion, and metastasis of Huh-7 cells. IL-6 treatment affected the expression of miRNAs, and miR-603 was overexpressed in HCC tissues. Also, miR-603 overexpression promoted the proliferation, migration, invasion, and metastasis of Huh-7 cells. Bioinformatic analysis predicted that miR-603 targets the 3′-UTR region of FABP1. However, miR-603 overexpression inhibited the expression of the FABP1 but increased the CPT1A, PPAR-*α*, and SREBP1 expressions. FABP1 overexpression reduced ROS in HCC cells, while miR-603 can reverse these effects.

**Conclusion:**

Our results indicate that in the pathogenesis of HCC, IL-6 induces miR-603 expression, which subsequently inhibits FABP1 expression, promotes the lipid metabolism- and synthesis-related proteins, and finally increases the cellular oxidative stress level and leads to the metastasis of HCC.

## 1. Introduction

Hepatocellular carcinoma (HCC) is the second leading cause of cancer-related death worldwide [1]. The invasiveness and metastatic potential are two key factors affecting the prognosis of patients with HCC, yet the mechanism remains largely unknown [2]. In terms of dietary structure, lipid metabolism homeostasis plays a central role in preventing HCC, yet its breakdown is a key risk factor associated with the development and progression of HCC [3]. The synthesis, transport, and catabolism of fatty acids are regulated by fatty acid-binding proteins (FABPs), which function as sensors to regulate the homeostasis of lipid metabolism. There are 12 members of the FABP family, which are primarily involved in peripheral free fatty acid (FFA) uptake, transport, fatty acid synthesis, lipid processing, storage, oxidative decomposition, and output.

The liver plays an essential role in fatty acid metabolism, and abnormal lipid metabolism is closely associated with the pathogenesis of nonalcoholic fatty liver disease (NAFLD) and HCC [4, 5]. FABP1 is a special FABP primarily found in the liver, which has two fatty acid-binding sites and a strong affinity for fatty acids. FABP1 is overexpressed in the cytoplasm of HCC cells [6]. microRNA (miRNA) is an endogenous noncoding RNA that participates in the negative regulation of target genes by binding to the 3′-UTR (i.e., untranslated region) of mRNA. Studies have shown that miRNAs play important roles in the regulation of lipid metabolism [7, 8].

Specifically, miRNAs are involved in the regulation of lipid metabolism-related proteins, thus playing vital roles in the development and progression of HCC [9–11]. The transcription and expression of miRNAs are regulated by a variety of inflammatory cytokines [12]. IL-6 is an important cytokine in the pathogenesis of HCC [13]. However, it is unknown whether IL-6 can regulate FABP1 and related miRNAs during the development of HCC. In the current study, we systematically investigated the role of the IL-6/miR-603 signaling in regulating the pathogenesis of HCC by acting on FABP1, thereby providing new perspectives on the pathogenesis and treatment of HCC.

## 2. Materials and Methods

### 2.1. HCC Tissue Specimens

A total of 18 pairs of HCC and adjacent healthy tissues were obtained from patients of Wuxi People's Hospital (Wuxi, China), who were diagnosed with HCC and received surgery between July 2016 and September 2018. The patients have not received any chemotherapy before the surgery. The experiment was approved by the Ethics Committee of Nanjing Medical University. Informed consent was obtained from all patients.

### 2.2. Tissue Array

The tissue array chip was purchased from Shanghai Zuocheng Biotechnology Co., Ltd. (China). The chip contained 90 cases of HCC tissues with corresponding adjacent tissues.

### 2.3. Immunochemistry

Tissue specimens were sliced at 4-5 *μ*m. Antigen retrieval was performed with a citrate buffer (pH 6.0) at 90°C. The slides were blocked with goat serum (Beyotime Biotechnology, Shanghai, China) before the primary FABP1 antibody was added at a ratio of 1 : 100 (Sigma-Aldrich, St. Louis, MO, USA). After incubating overnight at 4°C, the slides were incubated with goat anti-rabbit secondary antibody (CWBio, Beijing, China) and developed with 3,3-diaminobenzidine (DAB) (Beyotime Biotechnology, Shanghai, China). Hematoxylin (Servicebio, Wuhan, China) was used for nuclear counterstaining.

To evaluate the stained tissues, ten fields of view were randomly selected under 400x magnification, and 200 cells were randomly picked from each field. The staining intensity (*A*) was defined as no stain (one point), mild positive staining (light yellow, two points), moderate positive staining (brown or dark yellow, three points), or strong positive staining (tan or dark brown, four points). The percentage of positive cells (*B*) was evaluated based on the following: *B* < 5% was 0 points, 5% ≤ *B* < 25% was one point, 25% ≤ *B* < 50% was two points, 50% ≤ *B* < 75% was three points, and *B* ≥ 75% was four points. The staining index = *A* × *B*. All histopathological sections were read, diagnosed, and recorded by two senior pathologists with more than 3 years of combined experience.

### 2.4. Oil Red O Staining of Liver Tissues

The solution was made by mixing 6 ml Oil Red O (Sigma-Aldrich, St. Louis, MO, USA) stock solution with 4 ml distilled water. After mixing for 10 min, the staining buffer was added dropwise onto the tissue for a 5-10 min incubation period. Excess staining buffer was removed with a solution of 60% Oil Red O and isopropanol. The tissues were washed with distilled water and counterstained with hematoxylin (Servicebio, Wuhan, China).

### 2.5. Quantitative Polymerase Chain Reaction (qPCR)

Total RNA was extracted from tissues according to the TRIzol protocol (Invitrogen, Carlsbad, CA, USA). First, 1 *μ*g of total RNA was used to generate cDNA according to the PrimeScript RT kit (TaKaRa, Shiga, Japan). Next, 2 *μ*l of cDNA was used in the real-time qPCR system. U6 was used as the internal reference, and the relative expression of miR-603 was calculated with the 2^−ΔΔCt^ method. Each experiment was repeated in triplicate.

### 2.6. Cell Culture, Induction, and Microarray Analysis

The human hepatocyte cell lines, Huh-7 and HepG2, were purchased from Cell Bank of Shanghai Institute of Life Sciences, Chinese Academy of Sciences (Shanghai China). Cells were cultured in high-sugar DMEM medium (Gibco, Gaithersburg, MD, USA) with 10% fetal bovine serum (FBS, Biological Industries, Cromwell, CT, USA) with 100 U/ml streptomycin and 100 U/ml penicillin (HyClone Company, Logan, UT, USA) in a 37°C humidified incubator with 5% CO_2_.

For IL-6 treatment experiments, the Huh-7 cells were treated with 10 ng/ml IL-6 (Sigma-Aldrich, St. Louis, MO, USA) for 72 h. After the incubation, total RNA or protein was collected for miRNA array or Western blot detections.

The total RNA of Huh-7 cell was extracted, and the miRNA microarray analysis was performed by Guangzhou Ribobio Co., Ltd. (Guangdong Province, China). One microgram of total RNA was added into a nuclease-free RNA sample PCR tube, and the small RNAs (<300 nucleotides) were separated using the MilliporeSigma Centriplus Centrifugal Concentrators Microcon YM-100 (MilliporeSigma, Burlington, MA) and adding poly(A) to the 3′ end of the small RNA for hybridization at 37°C. After hybridization, dyes labeled with specific flash biotin were used, followed by scanning and analyzing the data by Affymetrix Gene Chip™ Command Console Software (AGCC).

### 2.7. Transfection

Huh-7 or HepG2 cells were seeded in six-well plates to reach about 70% confluency. For overexpression experiments, 50 nM miR-603 mimics (Ribobio, Guangzhou, China) and 5 *μ*l Lipofectamine® RNAiMAX (Invitrogen, Carlsbad, CA, USA) were added into the cells for 6 h in Opti-MEM medium (Gibco, Gaithersburg, MD, USA). A nonspecific mimic was used as a negative control (NSM). After 6 h of incubation, the medium was replaced with a complete medium for 72 h for subsequent experiments.

### 2.8. Lentiviral Infection

Huh-7 cells were seeded into six-well plates and allowed to reach 30% confluency. Approximately 60 *μ*l of HiTransG A (Genechem Co., Ltd., Shanghai, China), 4 *μ*l 7 × 10^8^ TU/ml LV-FABP1 lentivirus (Genechem Co., Ltd., Shanghai, China), and 4 *μ*l 8 × 10^8^ TU/ml FABP1 RNAi lentivirus (Genechem Co., Ltd., Shanghai, China.) were added into the 1.5 ml complete medium; the scramble sequence was used as a negative control (NC). After 12 h of incubation, the medium was replaced with complete medium for 72 h for subsequent experiments.

### 2.9. FFA Treatment and Oil Red O Staining of Cells

FFA was prepared with 1 : 2 ratio of palmitic acid (PA, Sigma-Aldrich, St. Louis, MO, USA) and oleic acid (OA, Sigma-Aldrich, St. Louis, MO, USA). The FFA was dissolved in 0.1% diethylpyrocarbonate- (DEPC-) treated water (HyClone Company, Logan, UT, USA) containing 0.1 mM NaOH and 1% BSA (Absin, Shanghai, China). OA and PA were stored at 20 mM and 10 mM at -20°C and dissolved in a 75°C water bath before use.

After treating the cells in 12-well plates, the cell supernatants were removed. Cells were washed three times with PBS (HyClone Company, Logan, UT, USA) and fixed with 4% neutral formaldehyde for 10 min. Next, the fixed cells were thoroughly washed with PBS and 60% isopropanol for 10 s. Oil Red O (Sigma-Aldrich, St. Louis, MO, USA) was added for 10 min and then washed with 60% isopropanol for 5 s. Finally, the cells were stained with hematoxylin (Servicebio, Wuhan, China) for 10 min, and the optical intensity was measured using a spectrophotometer at 500 nm (Thermo Fisher Scientific).

### 2.10. FABP1 Labelization and Colocalization

For enhanced green fluorescent protein- (EGFP-) labeled FABP1 protein induction, the cells were cultured in 24-well plates and treated with 175 *μ*g/ml EGFP-labeled FABP1 protein (FABP1-EGFP) (Public Protein/Plasmid Library, Nanjing, China), and the EGFP protein was used as control; 24 h later, the cells were treated with Golgi apparatus or lysosome tracker as followed.

Golgi apparatus tracker red (Beyotime Biotechnology, Shanghai, China) work fluid was prepared according to the manufacturer's protocol. Cells were washed with Hank's balanced salt solution with Ca2+ and Mg2+ (Beyotime Biotechnology, Shanghai, China) and incubated with Golgi-Tracker red work fluid at 4°C for 30 min, and then, the cells were washed with DMEM three times and incubated with DMED at 37°C for 30 min. Cells were observed under SP8 laser scanning confocal microscopy (Leica, Gaman), and the fluorescence intensity was measured by ImageJ software.

Lysosome tracker red (Beyotime Biotechnology, Shanghai, China) work fluid was prepared according to the manufacturer's protocol. Cells were incubated with lysosome tracker red work fluid at 37°C for 30 min; then, the cells were washed with DMEM for three times and incubated with DMED at 37°C for 30 min. Cells were observed under SP8 laser scanning confocal microscopy (Leica, Gaman), and the fluorescence intensity was measured by ImageJ software.

### 2.11. Western Blot Analysis

Huh-7 or HepG2 cells were collected in radioimmunoprecipitation assay (RIPA) (Beyotime Biotechnology, Shanghai, China) lysis buffer containing 1% phenylmethylsulphonyl fluoride (PMSF) (Beyotime Biotechnology, Shanghai, China) on ice for 10 min. The cell lysates were removed and placed into 1.5 ml Eppendorf tubes and centrifuged at 12,000 rpm for 15 min. Next, the supernatants were used to determine the total protein concentration using the bicinchoninic acid (BCA) (Beyotime Biotechnology, Shanghai, China) assay. Protein samples (25 *μ*g) were loaded into each well and separated with 5% concentrated gels and 12% separation gels. After blocking the membranes with 5% defatted milk, rabbit anti-human FABP1 (1 : 1000, SAB1410361, Sigma-Aldrich), mouse anti-human caspase 3 (1 : 1000, MAB10753 Sigma-Aldrich), mouse anti-human CPT1A (1 : 1000, ab128568, Abcam), rabbit anti-human PPAR-*α* (phospho S12, 1 : 1000, ab3484, Abcam), and rabbit anti-human SREBP1 (1 : 1000, ab191857, Abcam) antibodies were added and incubated at 4°C overnight. Next, the membranes were incubated with goat anti-rabbit (12348, Sigma-Aldrich) or goat anti-mouse (12349, Sigma-Aldrich) secondary antibody (1 : 5000) for 2 h at room temperature and developed with enhanced chemiluminescence (ECL) kit (Millipore, Burlington, MA, USA). The gels were imaged using the Syngene gel imager (Frederick, MD, USA).

### 2.12. Cell Proliferation

Huh-7 cells transfected with miR-603 mimics or the FABP1 overexpression lentivirus, along with the negative control group, were seeded in 96-well plates with 10^4^ cells/well, with three replicates in each group. After 24 h in complete medium, cell proliferation was detected with the Lights' EdU Apollo567 kit (RiboBio, Guangzhou, China), according to the manufacturer's protocol. The proliferation rate = (number of proliferated cells/total number of cells) × 100%.

### 2.13. Wound Healing Migration

Huh-7 cells transfected with miR-603 mimics or FABP1 overexpression lentivirus, along with the negative control groups, were seeded into six-well plates to a confluency of 80-90%. All experiment was performed in triplicate. First, a scratch was created by a 200 *μ*l tip at the bottom of the six-well plates. Next, a serum-free medium was added into the wells. Photos were taken every 12 h, beginning at 0 h for the control.

### 2.14. Reactive Oxygen Species (ROS) Detection

Cells were treated with FFA for 24 h in six-well plates. Next, 33 *μ*M dichlorodihydrofluorescein diacetate (DCFH-DA, Jiancheng Bioengineering Institute, Nanjing, China) was added into cells for 1 h. After the cells were collected by trypsinization and centrifugation at 5 min for 1000 rpm, the samples were read in a fluorescence microplate reader. Each sample was measured three times.

### 2.15. Statistical Analysis

Data were presented as the mean ± standard deviation (SD). Comparisons between counted data were performed using the homogeneity test of variance and independent sample *t*-tests or nonparametric tests. Kaplan-Meier plots were used for survival analysis. *P* values < 0.05 were considered statistically significant. The analyses were performed using SPSS 21.0 software (IBM, Chicago, IL, USA).

## 3. Results

### 3.1. FABP1 and Fatty Acid Expression in HCC and Adjacent Tissues

**Figure 1:**
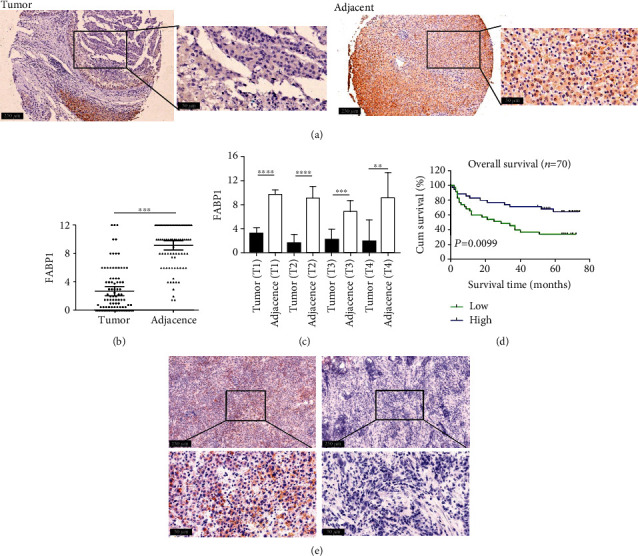
FABP1 expression in HCC and adjacent tissues. (a) Representative image of FABP1 staining by IHC in HCC tissue and the corresponding adjacent tissue array. The images of low-power field of view were magnified 100-fold, as shown in the left panel, scale bar = 250 *μ*m, and the images in the boxes were magnified 400-fold, as shown in the right panel, scale bar = 50 *μ*m. The FABP1-positive tissue was stained in brown. (b) FABP1 staining index in the tissue array. Data are expressed as the means ± SD, *n* = 90 for each group. ^∗∗∗^*P* < 0.001. (c) FABP1 staining score in four clinical stages of HCC and adjacent tissues. Data are expressed as the means ± SD, *n* = 37 for T1, *n* = 28 for T2, *n* = 15 for T3, and *n* = 10 for T4. ^∗∗^*P* < 0.01, ^∗∗∗^*P* < 0.001, and ^∗∗∗∗^*P* < 0.0001. (d) Survival curve generated by the Kaplan-Meier analysis in patients with high and low FABP1 expression (*n* = 70). (e) Representative image of fatty acid staining by Oil Red O staining in HCC tissue and the corresponding adjacent tissues. Images in the upper panel were magnified 100-fold, scale bar = 250 *μ*m. The images in the boxes were magnified 400-fold, as shown in the bottom panel, scale bar = 50 *μ*m.

Immunohistochemistry revealed that FABP1 expression is significantly lower in HCC tissues, as compared with adjacent tissues in nearly 90% (81/90) of cases (Figures 1(a) and 1(b)). HCC was further classified using the TNM stage according to the postoperative pathological features. Independent of the TNM stage, FABP1 expression was always lower than that of adjacent tissues (Figure 1(c)). Next, the correlation between FABP1 expression and overall survival was investigated. In the adjacent tissues, FABP1 expression was significantly correlated with overall patient survival (*P* < 0.01), as shown in Figure 1(d). Next, Oil Red O staining was used to check the lipid content in HCC and adjacent liver tissues. The adjacent tissues had an abundance of lipid, while lipid content in HCC tissues was low (Figure 1(e)).

### 3.2. Mutual Interaction of FABP1 and FFA

**Figure 2:**
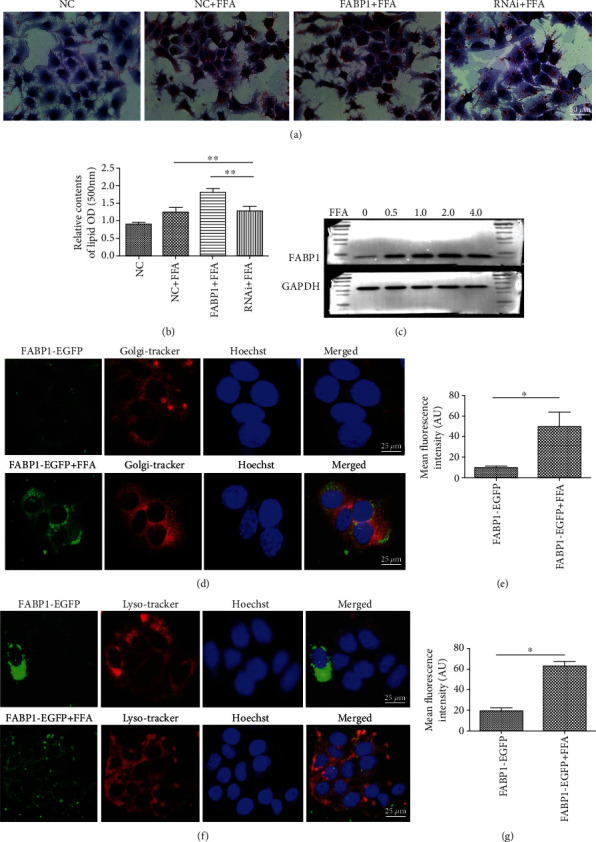
Interactions between FABP1 and FFA. (a) FABP1 increases the uptake of FFA by Huh-7 cells. The lipid content in the cells increased significantly after adding FFA in the culture medium compared with the control group (NC+FFA). Red color represents the lipid content. When the cells were transfected with the FABP1 overexpression lentivirus and then cultured with FFA (FABP1+FFA), the lipid content increased in the cells, while lipid content decreased significantly after FABP1 RNA interference (RNAi) transfection (FABP1-RNAi+FFA). The lipid was stained in red; 10 randomly selected fields were checked under an inverted microscope, bar = 50 *μ*m. (b) Quantification of the content of lipid in the cells. The relative content of lipid was measured by spectrophotometer at 500 nm (fold change from NC). Data are expressed as the means ± SD, *n* = 3 in each group. ^∗∗^*P* < 0.005. (c) FFA increases the uptake of FABP1 by Huh-7 cells. Expression of the FABP1 protein in Huh-7 cells after FFA treatment. Huh-7 cells were induced by FFA at different concentrations (0, 0.5, 1.0, 2.0, and 4.0 mM), and the expression of FABP1 was detected at different induction concentrations by Western blot. GAPDH was used as the internal loading control. (d, f) Colocalization of FABP1 with Golgi apparatus and lysosome. Cells were transfected with FABP1-EGFP vector (green) alone or induced with FFA after (FABP1-EGFP+FFA) transfection, and then, the cells were stained with Golgi-Tracker or Lyso-Tracker. The Golgi apparatus or lysosome was stained in red, and the nuclear was stained by Hoechst in blue. Cells of 10 randomly selected fields were observed by a confocal microscopy (Olympus, Tokyo, Japan), and the fluorescence intensity was calculated by ImageJ software. Bar = 25 *μ*m. (e, g) Quantification of the green fluorescence intensity in the cells and the bar graph was drawn. Data are expressed as the means ± SD, *n* = 3 in each group. ^∗^*P* < 0.05. Arbitrary units (AU).

From the data above, it was found that both FABP1 and lipid content in the HCC tissues were lower than adjacent tissues, because FABP1 has a strong affinity for fatty acids, so in order to study the interaction between FABP1 and FFA in HCC cells, the Huh-7 cells were transfected with the FABP1 overexpression lentivirus and then FFAs were added to the cells. Oil Red O staining revealed a large number of lipid that was stained in red, indicating that fatty acid-induced lipid accumulation is enhanced. Meanwhile, after infection of the Huh-7 cells with FABP1 RNAi, fatty acid-induced lipid content was reduced (Figures 2(a) and 2(b)). Moreover, the FFA can accelerate the uptake of the FABP1 protein in the Huh-7 cells in a FFA dose-dependent manner (Figure 2(c)). Furthermore, in order to verify that the FFA can accelerate Huh-7 cells to uptake the FABP1 protein, the FABP1 protein was colocalized with organelles. When the locations of Golgi apparatus and lysosome in the cells were compared, it was showed that the cells induced by FFA had more green fluorescence intensity in the cells; in another words, FFA was shown to promote the uptaking of FABP1 by Huh-7 cells (Figures 2(d)–2(g)).

### 3.3. FABP1 Promotes Apoptosis and Inhibits the Proliferation, Invasion, and Metastasis of HCC Cells

**Figure 3:**
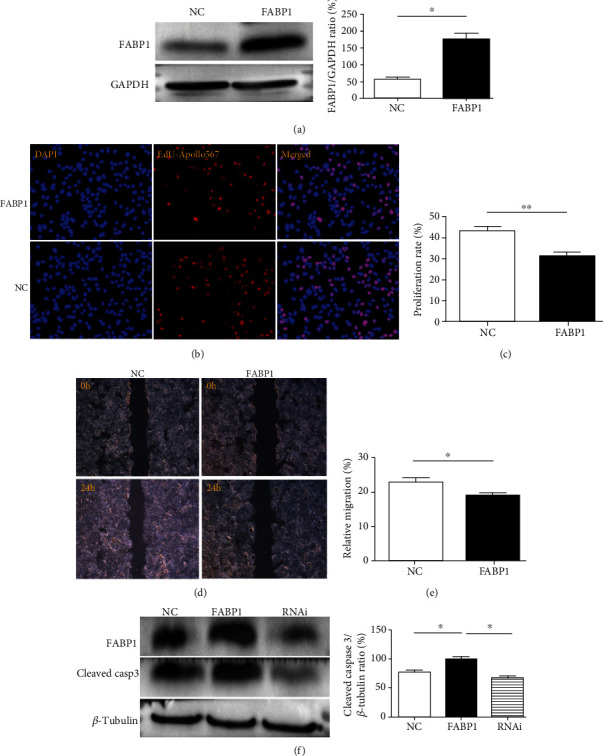
FABP1 inhibits the proliferation and migration of liver cancer cells and promotes apoptosis. (a) Western blot results of FABP1 overexpression in Huh-7 cells. Huh-7 cells were transfected with the FABP1 lentivirus (FABP1), and the blank lentivirus (NC) was used as control. Quantification of the Western blot (right panel). Data are expressed as the means ± SD, *n* = 3 in each group. ^∗^*P* < 0.05. (b) Huh-7 cell proliferation after FABP1 overexpression. The nuclear was stained by DAPI in blue; the proliferative cells were stained by EdU-Apollo567 in red. 10 randomly selected fields were checked under a fluorescence microscope. (c) Quantification of the proliferation rate (%). The proliferation ratio was calculated as the number of proliferating cells/total number of cells × 100% by ImageJ software. Data are expressed as the means ± SD, *n* = 3 in each group. ^∗∗^*P* < 0.01. (d) Huh-7 cell migration showed by the scratch assay after FABP1 overexpression; 10 randomly selected fields were checked under an inverted microscope. (e) Quantification of the migration rates (%). Data are expressed as the means ± SD, *n* = 3 in each group. ^∗^*P* < 0.05. (f) Huh-7 cell apoptosis after FABP1 overexpression or knockdown. The FABP1 overexpression or RNAi lentivirus was used for infection. Quantification of Western blot was shown in the right panel. Data are expressed as the means ± SD, *n* = 3 in each group. ^∗^*P* < 0.05.

To investigate the role of FABP1 in HCC cells, Huh-7 cells were infected with FABP1 overexpressing lentivirus. The infection efficacy was determined by Western blot analysis (Figure 3(a)). After 48 h, the proliferation rate of the FABP1 overexpression group is significantly lower than that of the negative control (NC) group (*P* < 0.01, Figures 3(b) and 3(c)). The wound healing experiments showed that FABP1-overexpressed Huh-7 cells had reduced migration abilities *in vitro* (*P* < 0.05, Figures 3(d) and 3(e)). In the presence of FFA, cleaved caspase 3 expression is increased by FABP1 overexpression and decreased by FABP1 silencing (RNAi), as shown in Figure 3(f).

### 3.4. IL-6 Promotes the Expression of miR-603, and miR-603 Overexpression Promotes the Proliferation and Metastasis of HCC Cells

**Figure 4:**
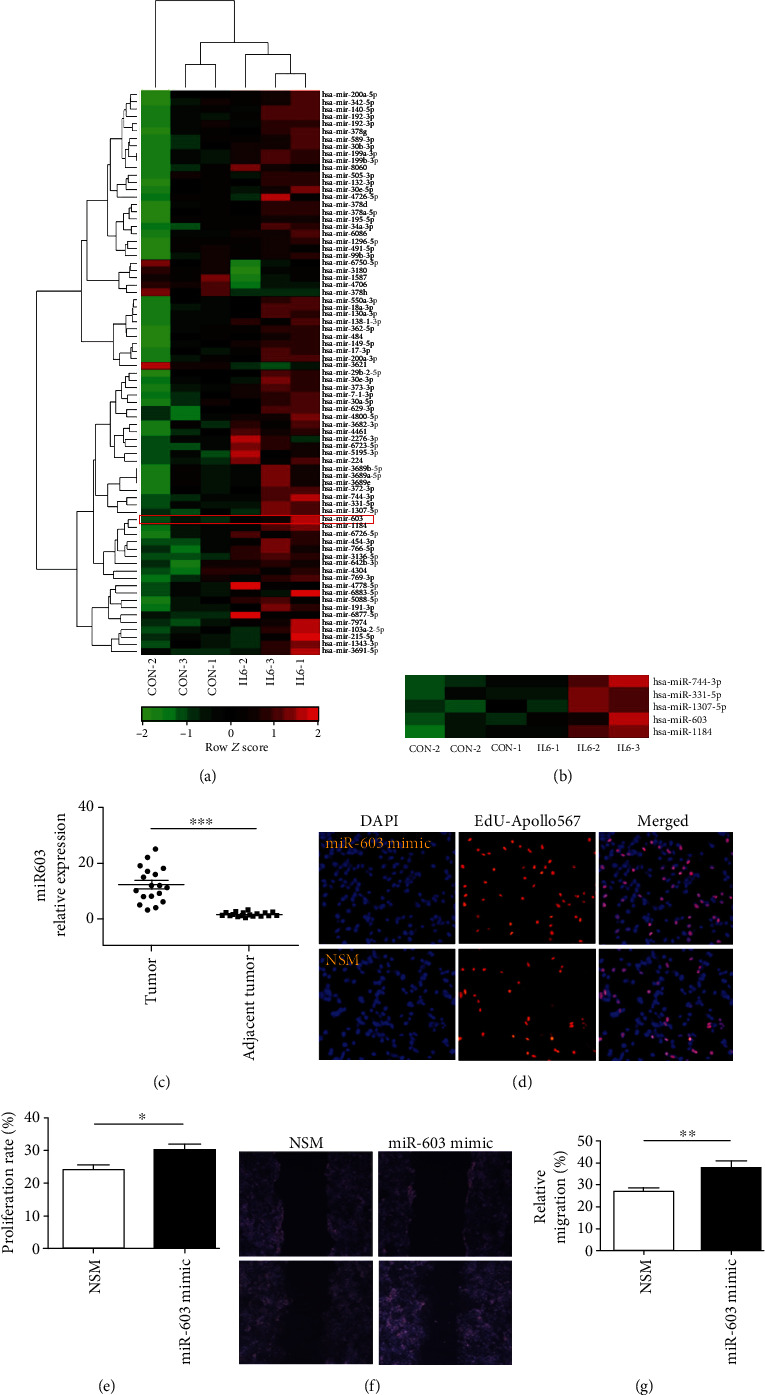
IL-6 induces the expression of miR-603, which promotes the proliferation and migration of HCC cells. (a) miRNA array after IL-6 treatment in Huh-7 cells. Differentially expressed miRNAs were profiled in three cases of DEPC water induction (CON-1, CON-2, and CON-3) and IL-6 induction (IL6-1, IL6-2, and IL6-3) using miRNA microarrays. A total of 77 deregulated miRNAs in the IL-6 treatment group (*P* < 0.01). The miR-603 was marked in red square. (b) miR-603 was higher in the IL-6 treatment group than in the control groups (*P* < 0.01). The top bar represents miRNA expression levels from -2 (green) to +2 (red). The individual identity of significantly deregulated miRNAs is shown on the right. (c) miR-603 expression levels in HCC and adjacent tissues. Data are expressed as the means ± SD, *n* = 18 in each group. ^∗∗∗^*P* < 0.001. (d) Cell proliferation after miR-603 overexpression. The miR-603 mimic or nonspecific mimic (NSM) was used to transfect cells, while nuclei were stained with DAPI in blue. Proliferative cells were stained with EdU-Apollo567 in red. 10 randomly selected fields were checked under a fluorescence microscope. (e) Quantification of the proliferation rate (%). The proliferation ratio was calculated as the number of proliferating cells/total number of cells × 100% by ImageJ software. Data are expressed as the means ± SD, *n* = 3 in each group. ^∗^*P* < 0.05. (f) Huh-7 cell migration showed by the scratch assay after FABP1 overexpression. (g) Quantification of the migration rate (%). 10 randomly selected fields were checked under an inverted microscope. Data are expressed as the means ± SD, *n* = 3 in each group. ^∗∗^*P* < 0.01.

To examine the function of IL-6 in HCC cells, Huh-7 cells were treated with IL-6 for 72 h, and RNA was analyzed using the miRNA chip. The result showed that 71 miRNAs were upregulated, and 6 were downregulated after IL-6 treatment (Figure 4(a)). These altered miRNAs were further validated in 18 pairs of HCC and adjacent tissues using qPCR, which showed that miR-603 expression was significantly upregulated in HCC tissues (Figures 4(b) and 4(c)). Next, Huh-7 cells were transfected with the miR-603 mimic (miR-603 overexpression) and nonspecific mimics (NSM). Cell proliferation was detected 48 h after transfection and revealed that miR-603 overexpression promoted the proliferation of HCC cells (Figures 4(d) and 4(e)). The scratch migration assay showed that miR-603 could promote the migration abilities of HCC cells (Figures 4(f) and 4(g)). ^∗^*P* < 0.05 and ^∗∗^*P* < 0.01.

### 3.5. IL-6 and miR-603 Block FABP1 Expression and Regulate ROS Levels in HCC Cells

**Figure 5:**
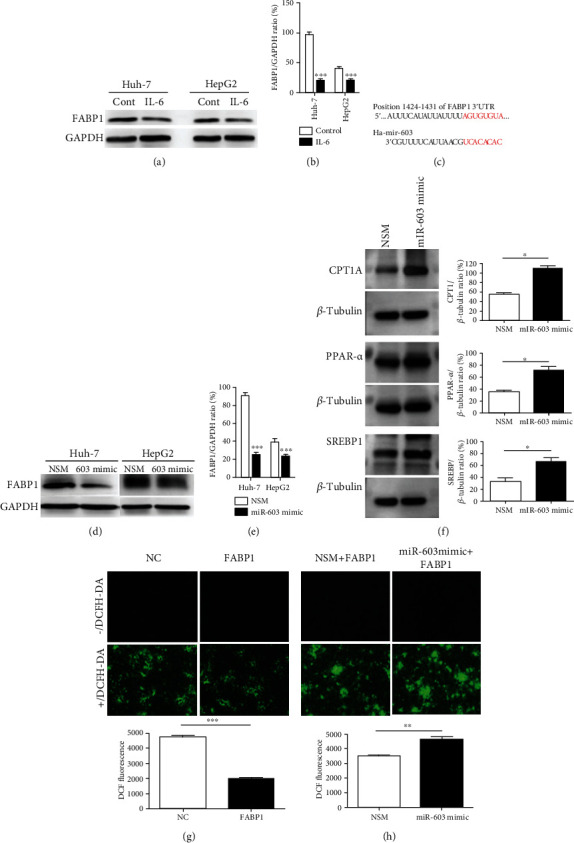
FABP1 reduces ROS levels in HCC cells, while miR-603 reverses the inhibitory effect of FABP1 on oxygen free radicals. (a) Western blot results of FABP1 expression level after IL-6 treatment in Huh-7 and HepG2 cells. (b) Quantification of the Western blot. Data are expressed as the means ± SD, *n* = 3 in each group. ^∗∗∗^*P* < 0.001. (c) The binding sites of FABP1 mRNA 3′-UTR (1424 to 1431) to miR-603 were shown as red. (d) Western blot results of FABP1 expression level after miR-603 treatment in Huh-7 and HepG2 cells. (e) Quantification of the Western blot. Data are expressed as the means ± SD, *n* = 3 in each group. ^∗∗∗^*P* < 0.001. (f) Western blot results of lipid metabolism- and synthesis-related protein expression after miR-603 mimic transfection in Huh-7 cells. Quantification of the Western blot was shown in the right panel. Data are expressed as the means ± SD, *n* = 3 in each group. ^∗^*P* < 0.05. (g) ROS levels after FABP1 overexpression treatment. Cells transfected with the FABP1 overexpression lentivirus or NC were stained with DCFH-DA (green), and 10 randomly selected fields were checked under a fluorescence microscope. Quantification of the DCF fluorescence was shown in down panel. Data are expressed as the means ± SD, *n* = 3 in each group. ^∗∗∗^*P* < 0.001. (h) ROS levels after miR-603 treatment. Cells were transfected with the FABP1 lentivirus or NC. Next, the cells were transfected with miR-603 (FABP1+miR-603 mimic) or NSM (FABP1+NSM) and stained with DCFH-DA; 10 randomly selected fields were checked under a fluorescence microscope. Quantification of the DCF fluorescence was shown in down panel. Data are expressed as the means ± SD, *n* = 3 in each group. ^∗∗^*P* < 0.01.

The function of miR-603 in regulating FAPB1-related ROS levels was evaluated. In both Huh-7 and HepG2 cells, FABP1 was downregulated after 72 h of IL-6 treatment (Figures 5(a) and 5(b)). The target gene analysis predicted that the FABP1 3′-untranslated region (3′-UTR region) 1424-1431 has eight binding sites for miR-603 (Figure 5(c)). Overexpression of the miR-603 inhibited FABP1 protein expression in both Huh-7 and HepG2 cells (Figures 5(d) and 5(e)). In order to check the roles of miR-603 in other lipid metabolism- and synthesis-related proteins, the Western blot was performed. The results showed that overexpression of miR-603 can increase the CPT1A, PPAR-*α*, and SREBP1 protein expressions (Figure 5(f)). In addition, the role of FABP1 and miR-603 in the ROS levels of Huh-7 cells was investigated; it was found that overexpression of FABP1 can reduce the ROS levels in Huh-7 cells (Figure 5(g)), which could be reversed by miR-603 overexpression (Figure 5(h)).

**Figure 6:**
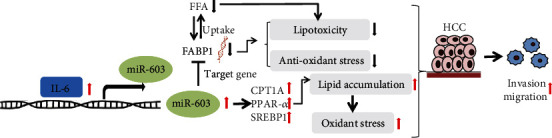
The signaling pathway diagram regulated by miR-603/FABP1 in HCC invasion and metastasis. The activation role was indicated by the black arrow, and the inhibition role was indicated by the suppress symbol; the red arrow was used to indicate the upregulation, and the black downward arrow was used to indicate the downregulation.

In summary, the results indicate that IL-6 induces miR-603 expression, which can subsequently inhibit the expression of FABP1 and increase the expression of CPT1A, PPAR-*α*, and SREBP1, thus enhances intracellular oxidative stress, thereby promoting the invasion and metastasis of HCC (Figure 6).

## 4. Discussion

Disorders of lipid metabolism play central roles in the development and progression of HCC. Studies have shown that FABP1 expression is significantly increased in steatohepatitis. Overexpressed FABP1 results in the accumulation of intracellular FFA and increases cell lipid toxicity. By inhibiting FABP1 expression, NAFLD-related damage can be improved [14]. The Western food diet in FABP1^−/−^ knockout mice showed preventive effects against obesity and steatohepatitis [15]. Although an earlier study shows that FABP1 was overexpressed in HCC tissues compared to adjacent liver tissues [16], another study found that FABP1 is dysregulated in HCC and that patients with low FABP1 expression have a lower degree of tumor differentiation [17]. In addition, another study showed that 47% (76/163) HCCs exhibited weak or even no immunoreactivity of FABP1 and low FABP1 expression has been associated with poorer prognoses of patients [18]. These latter studies are consistent with our current findings and the discrepancies with the study from Ku et al. could be due to differences in patient demographics between the studies. Specifically, in the current study, we found that FABP1 expression was lower in HCC tissues than adjacent tissues. Further analysis revealed that FABP1 expression in HCC tissues at different clinical stages was also lower than that in the adjacent tissues. Patients with high expression of FABP1 in adjacent tumor tissues were more likely to have longer survival times. In order to better understand the role of FABP1, we are continuing to collect the HCC samples and will check the FABP1 expression in a larger sample size.

FABP1 and fatty acid expression in HCC that is downregulated might be due to the significant enhancement of de novo fatty acid synthesis in tumor cells. Approximately 93% of the fatty acids of triglycerides contained in tumor cells are produced by de novo fatty acids [19]. Therefore, although downregulation of FABP1 expression reduces cellular fatty acid uptake and metabolic capacity, in the context of abnormal activation of de novo fatty acid synthesis in tumor cells, the downregulated expression may produce protective effects and possibly avoid the lipotoxicity caused by intracellular lipid accumulation.

FABPs present in the tumor microenvironment can regulate the development and pathogenesis of tumors [20] and may be used as potential targets for anticancer drugs [21]. FABPs, which are secreted into extracellular fluids, may also be used as a diagnostic marker of organ damage [22]. For example, FABP1 secreted into serum can be used as a biomarker of liver injury [23]. Our previous study reported that FABP4 derived from adipocytes in the microenvironment could promote the metastasis of cholangiocarcinoma by entering into cancer cells [24]. In the current study, the FABP1 protein colocalization, Oil Red O staining, and Western blot assays demonstrated that FFA promoted the uptake and expression of FABP1 by HCC cells. Simultaneously, FFA uptake was increased by HCC cells that overexpressed FABP1. In conclusion, we speculate that FABP1 in the HCC microenvironment may regulate the distribution of fatty acids by freely entering and exiting HCC cells, thereby regulating tumor progression.

IL-6 plays a key regulatory role in the pathogenesis of many types of cancer [25]. We have previously reported on elevated IL-6 levels in HCC [26, 27]. The current study found that IL-6 downregulated FABP1 expression in HCC cells. miRNAs are endogenous noncoding RNAs that participate in the negative regulation of target genes by binding to the 3′-UTR of mRNA. IL-6 can regulate the development of tumors by promoting the expression of specific miRNAs [28], and miRNAs can improve hepatic steatosis and injury by inhibiting the expression of FABP1 [29]. In this experiment, the miRNA array was used to detect miRNA alterations after IL-6 treatment. The miRNA expression profile in HCC cells changed significantly after IL-6 induction. Five upregulated miRNAs, including miR-603, were further verified by qPCR using human HCC and adjacent tissues. Bioinformatic target genes predicted that miR-603 has a direct binding site to the 3′-UTR of FABP1. Western blot analysis showed that miR-603 overexpression could reduce the expression of FABP1. The role of miR-603 as a tumor suppressor gene has been reported in many other studies [29, 30]. Our *in vitro* functional studies have shown that miR-603 promotes the proliferation and migration of HCC cells. In the pathogenesis of HCC, high levels of IL-6 likely promote the expression of miR-603. In return, miR-603 blocks the expression of FABP1, thereby promoting the development and progression of HCC.

Except for the FABP1, we also check the roles of miR-603 in other lipid metabolism- and synthesis-related proteins. CPT1A is a rate-limiting enzyme in the transport of long-chain fatty acids for *β*-oxidation [31], PPAR-*α* is known to have an important role in fatty liver, and the mechanism of carcinogenesis has been clarified [32]. SREBP1 is crucial for lipogenesis as well as HCC cell proliferation and metastasis [33]; in the current study, it was found that the expressions of these three proteins above were increased in HCC cells transfected by miR-603 mimic; the results indicated that miR-603 can promote the lipid accumulation and then accelerate the HCC progression.

Imbalanced oxidative stress plays an important role in the occurrence and development of cancer [34]. Studies have found that FABP1 can exert antioxidative stress by capturing and removing ROS, thereby protecting the liver [35]. Decreased expression of FABP1 can increase cellular oxidative stress (OS) levels [36]. These studies supported our results; in our current study, *in vitro* cell culture experiments have shown that high expression of FABP1 can inhibit the proliferation and migration of HCC cells while promoting the apoptosis of HCC cells. Hence, FABP1 may function as a tumor suppressor. In addition, FABP1 overexpression can inhibit ROS levels in HCC cells, yet miR-603 can block this effect. Thus, we speculated that in the pathogenesis of HCC, IL-6 likely inhibits FABP1 expression through miR-603, thereby enhancing intracellular ROS levels and promoting the pathogenesis of HCC.

However, the experimental results of the current study need to be further verified with large sample size and in animal experiments. Why FABP1 expression is downregulated in HCC? What factors other than miR-603 can regulate FABP1 expression? What are the specific interaction pathways between FABP1 and FFA in the pathogenesis of HCC? Does IL-6 also regulate FABP1 expression through other pathways? And what is the relationship between FABP1 and other lipogenesis proteins? Does the miR-603 regulate the CPT1A, PPAR-*α*, and SREBP1 protein expressions through FABP1? All of these issues need to be further investigated in the future.

In summary, the results of this study provide new perspectives on the regulation of lipid metabolism in HCC, while also demonstrating the importance of further investigations into the role of IL-6 in the pathogenesis of HCC.

## Data Availability

Answer: Yes. Comment: The experimental data used to support the findings of this study are included within the article.
